# Visually inferring elasticity from the motion trajectory of bouncing cubes

**DOI:** 10.1167/jov.20.6.6

**Published:** 2020-06-09

**Authors:** Vivian C. Paulun, Roland W. Fleming

**Affiliations:** Department of Psychology, University of Gießen, Gießen, Germany; Department of Psychology, University of Gießen, Gießen, Germany

**Keywords:** material perception, elasticity, intuitive physics, motion perception

## Abstract

Visually inferring the elasticity of a bouncing object poses a challenge to the visual system: The observable behavior of the object depends on its elasticity but also on extrinsic factors, such as its initial position and velocity. Estimating elasticity requires disentangling these different contributions to the observed motion. We created 2-second simulations of a cube bouncing in a room and varied the cube's elasticity in 10 steps. The cube's initial position, orientation, and velocity were varied randomly to gain three random samples for each level of elasticity. We systematically limited the visual information by creating three versions of each stimulus: (a) a full rendering of the scene, (b) the cube in a completely black environment, and (c) a rigid version of the cube following the same trajectories but without rotating or deforming (also in a completely black environment). Thirteen observers rated the apparent elasticity of the cubes and the typicality of their motion. Generally, stimuli were judged as less typical if they showed rigid motion without rotations, highly elastic cubes, or unlikely events. Overall, elasticity judgments correlated strongly with the true elasticity but did not show perfect constancy. Yet, importantly, we found similar results for all three stimulus conditions, despite significant differences in their apparent typicality. This suggests that the trajectory alone contains the information required to make elasticity judgments.

## Introduction

In order to interact with objects, we need to anticipate their mechanical properties and likely future behavior. For example, to grasp an object, we must adjust our grip to its weight and surface friction (Paulun, Gegenfurtner, Goodale, & Fleming, 2016), and to catch a ball, we adjust the interception strategy to the ball's elasticity (Diaz, Cooper, Rothkopf, & Hayhoe, 2013). Because mechanical properties, such as elasticity, weight, or viscosity, have no optical correlate, the visual system has to estimate them, much as it has to estimate depth from the two-dimensional image. Generally, two different mechanisms come in to play here: an “associative route” and an “estimation route” (Fleming, 2014; Paulun, Schmidt, van Assen, & Fleming, 2017; Schmidt, Paulun, van Assen, & Fleming, 2017). First, for familiar materials, the visual system can identify the material from its appearance and recall the associated mechanical properties from memory. For example, images of fabric are rated soft, whereas images of metal are rated hard and cold (Fleming, Wiebel, & Gegenfurtner, 2013; Schmidt et al., 2017). Second, the visual system can estimate mechanical properties of even unfamiliar materials through diagnostic visual cues in the image or motion sequence. For example, the shape and motion pattern of a fluid are used to estimate its viscosity (Kawabe, Maruya, Fleming, & Nishida, 2015; Paulun, Kawabe, Nishida, & Fleming, 2015; van Assen, Barla, & Fleming, 2018), speed and curvature are cues to stiffness of bending objects (Norman, Wiesemann, Norman, Taylor, & Craft, 2007), and the deformation and motion flow are cues to stiffness of nonrigid objects (Kawabe & Nishida, 2016; Paulun et al., 2017) and of cloth (Bi & Xiao, 2016). Shape and motion cues can also interact with optical cues, for example, in case of nonrigid breaking materials (Schmid & Doerschner, 2018). Here we investigate the estimation of elasticity based on the way objects bounce and interact with their surroundings.

In physics, elasticity is the ability of a body to return to its original shape and size after a distorting force is removed. This can be on a macroscopic level but also—invisible to the naked eye—on a microscopic or even atomic level. A sponge, for example, is elastic and bounces back to its original shape when released. Playdoh, on the other hand, is not elastic and stays in a new shape after being deformed. Elasticity is not to be confused with the *elastic modulus*. In engineering, elastic materials are described by two measures: an elastic modulus and an elastic limit. The *elastic modulus* describes how much a material deforms as a function of the applied force (slope of the material's stress-strain curve). The *elastic limit* is the amount of force that, when exceeded, leads to a permanent deformation. Thus, the elastic modulus is related to how *soft* or *compliant* a material appears. This is not what we investigate in the present study but has been studied before (Kawabe & Nishida, 2016; Masuda et al., 2013; Norman et al., 2007; Paulun et al., 2017; Schmidt et al., 2017). Here, we investigate bouncing or colliding objects, which may have high elasticity even when they are seemingly rigid (e.g., in Newton's cradle). A *collision is perfectly elastic* when the total kinetic energy between the two bodies (e.g., a ball and the floor) remains constant. In this ideal case, a ball would infinitely bounce to the same height (assuming no air resistance). In an *ideal inelastic collision*, on the other hand, the total kinetic energy is converted into other forms, such as heat, vibration, or noise. In this case, the ball would not bounce back at all but land dead on the floor. In the real world, partially elastic collisions are the most common forms of collisions (i.e., part of the total kinetic energy remains), whereas the rest is converted into other forms of energy. This results in a series of bounces that decay over time, where the ratio of heights between bounces is determined by the elasticity. In this study, we vary the amount of energy that remains after a collision (i.e., how elastic the collisions are), also known as the *coefficient of restitution*. The more energy is retained, the higher an object will bounce. This is what we refer to as elasticity in this article.

A number of previous studies have considered how the visual system infers the elasticity of objects from observations of object motion. For example, Warren, Kim, and Husney (1987) pointed out that spatiotemporal “events” are necessary to visually estimate elasticity; it cannot be perceived in static images. This makes intuitive sense, especially in the case of objects for which we have no expectations. Observing a ball flying through the air does not reveal information about its elasticity; only when the ball bounces off the floor can we infer its internal properties. This example illustrates another important fact about such “bounce events”: The exact behavior of the object depends not only on its elasticity but also on other, external factors. For example, how high a ball bounces depends on its elasticity but also its velocity and the elasticity of the floor it is bouncing off. If the ball lands in sand, it will not bounce back, even if it is highly elastic. Thus, the computational challenge for the brain is to disentangle the different causal contributions of internal material properties and external forces. How does the visual system solve this task?

Warren and colleagues (1987) investigated this question by asking observers to rate the apparent elasticity of bouncing balls shown in a reduced display, consisting of white circles starting from varying heights and bouncing vertically off the ground (represented as a line). The amount of visual information was systematically varied by occluding different parts of the display. They identified several possible visual cues to elasticity. The strongest cue was the relative height of consecutive bounces. When this information was not available, observers relied on the duration of bounces instead. Velocity information alone, however, was only a weak cue to elasticity. Nusseck and colleagues (2007) confirmed these results in an experiment in which observers passively judged the elasticity of simulated and rendered balls bouncing from one side of the screen to another. Interestingly, in two different experiments, the same authors showed that relative height (and estimated elasticity) were not sufficient to make predictions about the balls trajectory in order to intercept it. Participants either had to observe larger portions of the trajectory to intercept the ball or would simply pursue a follow-catch strategy.

Twardy and Bingham (2002) investigated scenes of bouncing balls with regard to their naturalness. Their computer-generated stimuli varied in naturalness with regard to the ball's elasticity, which could be higher than natural (i.e., with an increase in energy following a bounce), and with regard to gravity, which could be higher or lower than on earth. They found that although only hyperelasticity and hypogravity scenes were judged to look unnatural, observers were actually able to discriminate differences within all three types of manipulation. These results suggest that human observers are sensitive to changes in the spatiotemporal pattern (i.e., the trajectory) of bouncing balls. It is unclear how this relates to estimates of elasticity.

In the present study, we aimed better to understand visual elasticity perception in bouncing objects. Like the previous studies, we used computer simulations to create our stimuli as this allows maximal control over the parameter space. Unlike previous work, the simulated objects did not only bounce along one dimension (Warren et al., 1987) or two dimensions (Nusseck et al., 2007) but in a three-dimensional cubical room. Consequently, the objects could move in depth toward or away from the observer and could bounce off any of the walls or the ceiling, leading to more complex trajectories. We simulated a bouncing cube rather than a sphere, which expands the space of possible trajectories even further because the direction in which the cube travels after a bounce depends on its orientation (e.g., whether it hits the floor with an edge, a corner, or a side). Furthermore, the orientation of the cube during a bounce also influences how it continues to rotate, its rotational and translational velocity. We systematically varied the elasticity of the cube and, in addition, randomly varied its initial position, orientation, and velocity. In doing so, we were able to create several different trajectories for the same level of elasticity and thus effectively eliminated a direct mapping between elasticity and trajectory. Moreover, the initial speed and interactions with the surrounding walls also eliminate the direct mapping between elasticity and the relative height of consecutive bounces, the most important cue to elasticity suggested by previous literature. Here, we tested whether observers were still able to judge the elasticity of the cubes. Furthermore, to investigate which visual information was required to estimate elasticity, we systematically varied the amount of visual information. We either showed observers the full renderings of the scenes or only the cubes without the background. In the latter case, we also varied whether observers could see the cubes original motion (i.e., including deformations and rotations) or whether they saw a rigid, nonrotating cube following the same trajectory. This manipulation lets us investigate how much of the consequences of the bounce events observers have to see in order to make their elasticity judgments. In addition to the cube's elasticity, we asked observers to rate the typicality of the motion they observed. In doing so, we aimed to gain some insights about the canonical representations our observers have about bouncing objects and thus the information they use.

## Methods

### Participants

Thirteen students of the University of Gießen participated in the study, nine women and four men. Their average age was 26 years (*SD* = 5 years). All observers were naive with regard to the aims of the study and gave written informed consent prior to the experiment. The experiment took a maximum of 45 minutes and observers were compensated for their time with 6€ (i.e., 8€/hour). The experiment was approved by the local ethics committee (LEK FB06) and in accordance with the Declaration of Helsinki.

### Stimuli

Ninety computer renderings of a cubical object bouncing in a room served as stimuli. We chose a cubical object for two reasons: (a) Rotations of cubes are visible (unlike rotations of spheres) and (b) trajectories of cubes are more complex than those of spheres in the sense that the angle of reflection depends not only on the angle of incidence but also on the cube’ s orientation at the moment of collision. Thus, the space of possible trajectories is larger for cubical objects. In order to create the stimuli, we first simulated the physical behavior of the bouncy cubes and then rendered different versions of the same trajectory, systematically limiting the visual information present in each stimulus.

#### Simulation

**Figure 1.**
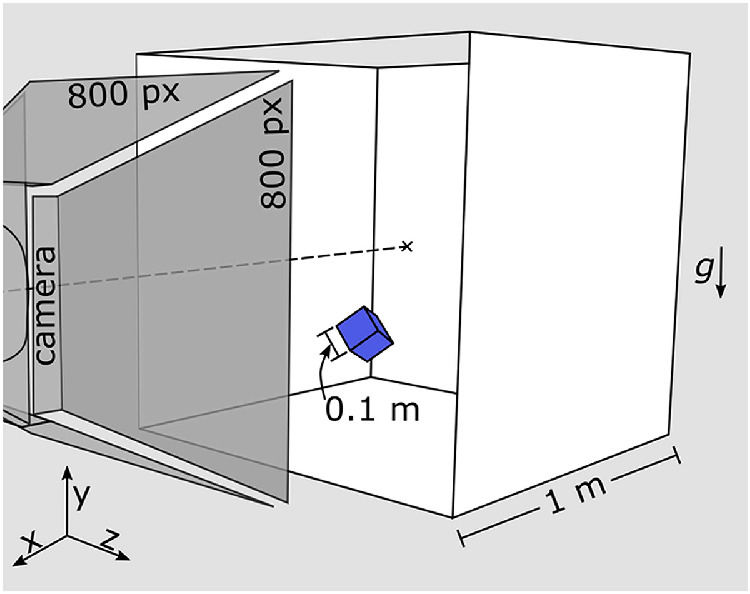
Sketch of the scene. A nonrigid cube (0.1 m edge length) was placed in a cubical room (1.0 m edge length) at a random position and orientation. The cube had a random initial velocity and additionally was subjected to gravitational force throughout the 2-s animation. The camera was positioned fronto-parallel to one side of the room. The “look-at” point (cross) was in the center of the room. The rendered images had a resolution of 800 × 800 pixels.

Stimuli were created with RealFlow 2014 (V.8.1.2.0192; Next Limit Technologies, Madrid, Spain), a dynamics simulation tool for three-dimensional computer graphics. The basic scene consisted of a cubical room with rigid walls of 1 m edge length (see Figure 1). Inside the room was a cubical target object with 0.1 m edge length. The cube was nonrigid (i.e., it could deform in a collision) because its internal stiffness parameters (*volume stiffness* and *length stiffness*) were both set to 0.5 (on a scale of 0.0 to 1,000.0). All other internal parameters (except elasticity) were kept constant at the default values (*mass*: 1.0 kg, *friction*: 0.3, *air friction*: 0.3, *internal damping*: 0.01). Gravity (9.81 m/s^2^), caused the cube to accelerate toward the floor. The elasticity of the cube was varied in 10 equal steps from 0.0 to 0.9 (on a scale from 0.0 to 1.0), describing the amount of energy that the cube retains when it collides. Basic tests of the behavior of simulated elastic objects can be found in the Appendix. For each level of elasticity, we created three different trajectories by randomly varying its initial position, orientation, and velocity. More specifically, the cube was randomly placed within the room so that its center of mass (CoM) was at least 0.2 m away from each wall. The initial orientation of the cube was set by rotating it three times, once about each axis *x*, *y*, and *z*. Each rotation angle was randomly set to a value between zero and π/2 (where an orientation of {0.0, 0.0, 0.0} describes a cube with the same orientation as the room). The initial velocity in each dimension was randomly set to a value between ±10 m/s. With these settings, we simulated the cube's trajectory for 61 frames at 30 fps (i.e., for 2.03 s). A given trajectory was not only determined by the level of elasticity of the cube but by nine external parameters: initial position (*x*, *y*, *z*), orientation (*x*, *y*, *z*), and velocity (*x*, *y*, *z*). Thus, we created 30 unique trajectories, three for each level of elasticity.

#### Rendering

**Figure 2.**
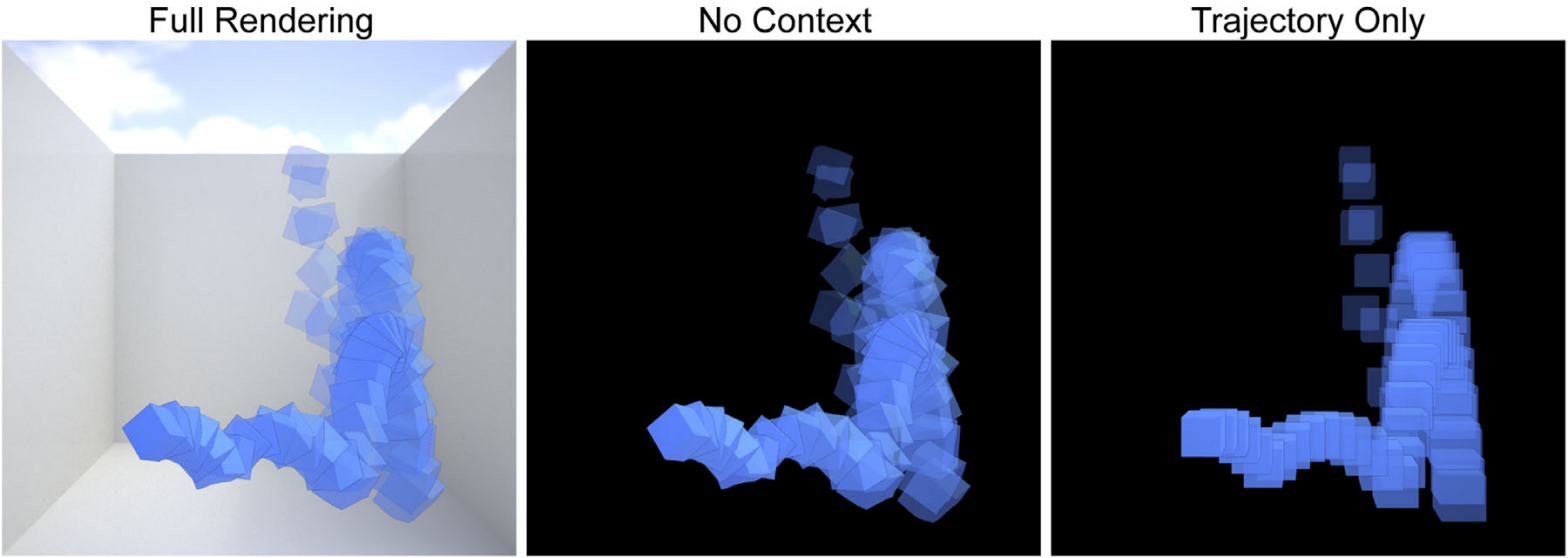
Stimuli of the three different conditions (Full Rendering, No Context, and Trajectory Only) for one example trajectory. Here, different time frames were made transparent and overlaid for visualization purposes. In the experiment, frames were presented at 30 fps to our participants. The same stimuli are shown in Supplementary Movie S1.

We rendered the scenes using RealFlow's built-in Maxwell renderer. The camera was positioned at central height in the scene, looking straight forward into the room (see Figure 1). The scene was illuminated with a high dynamic range illumination map of a beach scene (from the Maxwell Resource Library by Dosch Design, Marktheidenfeld, Germany). We rendered 61 PNG images (800 × 800 pixels) with a sampling level of 15. The cube was rendered with an opaque blue material. Each trajectory was rendered in three versions, controlling the amount of information present in each scene, that is, 90 stimuli in total (30 trajectories × 3 cue conditions); see Figure 2 and Supplementary Movie S1. In the *Full Rendering* condition, the walls of the room were rendered with a white matte material, except the ceiling (for illumination purposes) and the front wall (through which the camera looked), which were rendered completely transparent. Thus, in the *Full Rendering* condition, the target object and the room with which it was interacting were well visible to the observer (except the transparent ceiling). Additionally, the cube's cast shadow was visible in the *Full Rendering* condition, which probably helped observers interpret the three-dimensional position and motion of the cube (Kersten, Mamassian, & Knill, 1997). In the *No Context* condition*,* we did not render any of the walls and set the background intensity to 0.0, which made the background completely black. Thus, the *No Context* condition showed the exact same cube and trajectory as the full cube condition but on a completely black background i.e., all information about the room, the position of the walls, and the floor came only from the way the cube bounced. Finally, the *Trajectory Only* condition was rendered in the same way (i.e., with a black background), but instead of rendering the cube of the original simulation, we rendered a cube with the same dimensions and material that followed the simulated trajectory without rotating or deforming. Thus, in this condition, the information about where and how the cube bounced off the walls was even more reduced. It is important to note that even in the Full Rendering and No Context conditions, participants might not have been able to detect the cube's deformation because it occurred very briefly for only a single frame, just as one cannot see the deformation of a bouncing tennis ball. It is therefore possible that the deformation was not visible in any condition. All images used in the experiment are available for download at https://doi.org/10.5281/zenodo.3275880.

### Setup and procedure

Stimuli were presented on a Dell LCD monitor (model U2412M; resolution 1,920 × 1,080 pixels; refresh rate 60 Hz). Participants were seated in front of the monitor, approximately 60 cm away; the stimuli were roughly 20 degrees of visual angle. Participants viewed the stimuli binocularly. Although this introduces potential cue conflicts (between the monocular information of a three-dimensional room and the binocular information of a flat surface), this would—if at all—have an unsystematic effect on our results. Participants were instructed that they would rate the elasticity of objects presented to them in short animations, as well as the typicality of their motion. Elasticity was defined to them as the property that distinguishes, for instance, a hacky sack (not elastic) from a bouncy ball (very elastic). They were further instructed that objects can move in many ways, some of which are more common in reality than others; they are *typical* rather than *atypical*. If they were unsure about what that meant, we further instructed them that this refers to whether they can imagine seeing this motion in reality and how frequent it would be. Additionally, participants were told that they could throw a ball in many different ways and each time it would move slightly differently; some of the motions would appear more typical. Following the instructions, participants were presented with six animations in a familiarization phase. The six stimuli covered the lowest and highest elasticity levels in all three viewing conditions. Observers could go through the randomized animations at a self-chosen pace; no response was required. The purpose of the familiarization was to show participants the range of stimuli used in the experiment and to anchor their response range. If the observer had no further questions, the main experiment began. After the familiarization phase, all participants were confident that they could complete the task. On each trial, the observer was presented with one stimulus. The animation was displayed at 30 fps centrally on the screen in a loop until the response was given. Thus, observers could sample as much information as they needed to give a response. One frame with a mask of pixelated grayscale noise followed each animation cycle so that the beginning of a new cycle was clearly separable from the end of the current cycle. Two rating bars were presented underneath the animation, one to rate the elasticity (“not elastic” to “elastic”) and one to rate the typicality of the motion (“typical” to “atypical”). Observers had to adjust a randomly positioned dot on the bar with the mouse to give their rating. They first rated elasticity and then typicality. Observers could take as much time as needed to give their responses and complete a trial. Each trial was repeated three times for each observer (i.e., 270 trials in total). Trials were presented in a pseudorandom order, so that the specific trajectory shown in trial *n* could not be presented in a different version in trial *n* + 1 (e.g., first as Full Rendering, then as Trajectory Only). The entire experiment was run in MATLAB (The MathWorks, Inc.) using the Psychophysics toolbox (Brainard, 1997; Kleiner et al., 2007; Pelli, 1997).

### Data analysis

Ratings were saved on a scale between 0.0 (not elastic/atypical) and 1.0 (very elastic/typical). Raw data from the experiment are available at https://doi.org/10.5281/zenodo.3275880. We tested the influence of physical elasticity and visual cues on the elasticity and typicality ratings with Bayesian model comparison (or Bayesian analysis of variance [ANOVA]) using the JASP software (JASP Team). We additionally tested whether the intraobserver variability of elasticity ratings (as an indicator of certainty) varies between cue conditions by calculating the standard deviation of ratings of the same stimulus by the same observer and then averaged across stimuli and across observers. We compared the three cue conditions with Bayesian paired samples *t* tests. In addition to Bayesian statistics, we report the corresponding results of standard null hypothesis testing in the Appendix because some readers might be more familiar with interpreting frequentist statistics. Importantly, standard and Bayesian hypothesis testing lead to the same conclusions.

## Results

### Elasticity ratings

**Figure 3.**
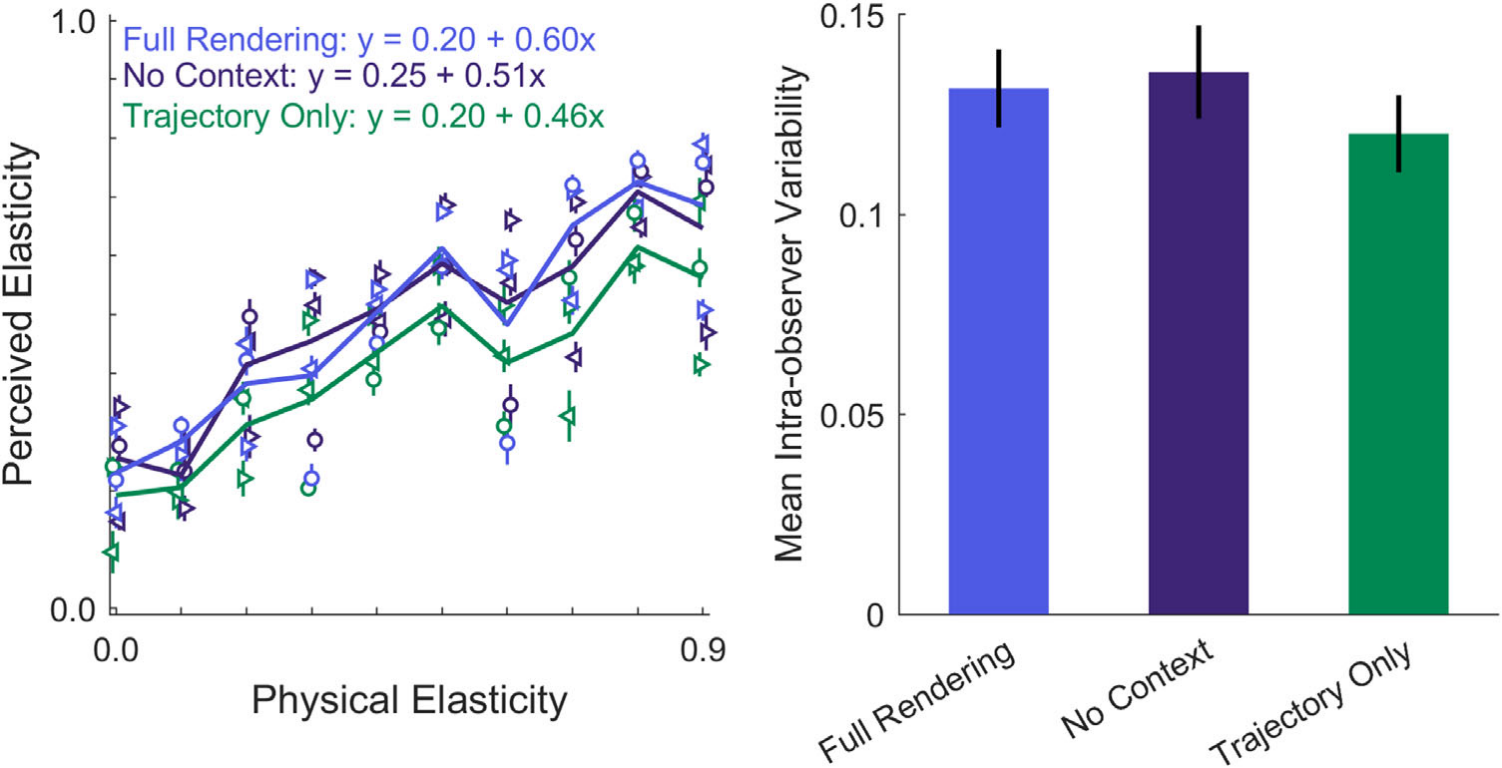
Results of the elasticity ratings. (A) Lines show average elasticity rating across trajectories and observers for each level of elasticity. Symbols show average across observers for different trajectories (i.e., three per level of elasticity). At each elasticity level, there are three symbols of each color: The same symbol represents the same trajectory, and the color represents the cue condition. Error bars show ± 1 *SEM* across participants. Markers of different conditions were slightly jittered along the *x*-axis to increase visibility. (B) Average intraobserver variability for different viewing conditions. We calculated the standard deviation of ratings of the same stimulus by the same observer and then averaged across stimuli and across observers. Error bars show ± 1 *SEM* across participants.

We analyzed both the elasticity and the typicality ratings with a Bayesian repeated-measures ANOVA. For that purpose, we compared four competing models to the null model (that no variable influences the rating) and evaluated the corresponding Bayes factors (BF): (1) a simple main effect model of *cue condition*, (2) a simple main effect model of *elasticity*, (3) a model with two main effects (*cue* + *elasticity*), and (4) a model with two main effects and an interaction. The results are summarized in Table 1. We found that Model 3 (i.e., two main effects and no interaction) could best explain the elasticity ratings. Specifically, BF_Cue + Elasticity_ = 7.67 * 10^73^, that is, the data are 7.67 * 10^73^ more likely under the hypothesis of two main effects than under H_0_, which is considered *extreme evidence for H_1_*(Wagenmakers et al., 2018). For comparison, the BF for either of the individual main effect models compared to the null model was lower (BF_Elasticity_ = 1.58 * 10^66^; BF_Cue_ = 95.62). Additionally, we found that the data were 73.61 times more likely under the model with two main effects than a model including main and interaction effects (BF_Cue + Elasticity_ / BF_Cue + Elasticity + Interaction_ = 73.61), which is considered *very strong evidence* (Wagenmakers et al., 2018). Thus, physical elasticity and cue condition independently influenced the elasticity ratings. Regardless of the cue condition, cubes that are more elastic were on average perceived to be higher in elasticity (see Figure 3A). This figure also indicates the equations of a linear fit between simulated and perceived elasticity for all trajectories. As can be expected (given that we did not find an interaction of the factors), a similar slope was found for the three cue conditions. Importantly, the pattern of ratings was the same in all three cue conditions; for example, if a specific trajectory was rated as much lower in elasticity than it actually was, then that trajectory was rated much lower in elasticity in *all* cue conditions (see, e.g., dip of all three lines at elasticity 0.6). Overall, however, the elasticity ratings were slightly lower in the *Trajectory Only* condition compared to the other two viewing conditions (BF_Full ≠ Rigid_ = 4.38 * 10^4^; BF_No Context ≠ Rigid_ = 6.41 * 10^5^). The rotations and deformations of the cube seemed to have enhanced the impression of elasticity in the other two conditions. To quantify this effect, we can compare the average ratings in the three conditions (averaged across elasticities). The Trajectory Only already accounts for 82.30% of the average rating in the Full Rendering condition. Adding rotations and deformations to the trajectory accounts for another 16.69% of the Full Rendering rating (i.e., average rating in No Context condition is 98.98% of the rating in the Full Rendering condition). The context information (i.e., the background in the full renderings) added only 0.02% to the average Full Rendering rating.

**Table 1.** Results of Bayesian repeated-measures ANOVAs.

Models	P(M)	P(M|data)	BF_M_	BF_10_	Error %
*Elasticity ratings*					
Null model (including subject)	0.200	1.286e –74	5.146e –74	1.000
Cues	0.200	1.230e –72	4.920e –72	95.617	0.781
Elasticity	0.200	2.033e –8	8.131e –8	1.580e +66	0.470
Cues + elasticity	0.200	0.987	294.454	7.670e +73	1.185
Cues + elasticity + cues * elasticity	0.200	0.013	0.054	1.042e +72	1.614
*Typicality ratings*					
Null model (including subject)	0.200	6.701e –63	2.681e –62	1.000	
Cues	0.200	1.998e –5	7.991e –5	2.981e +57	0.808
Elasticity	0.200	1.226e –62	4.902e –62	1.829	0.327
Cues + elasticity	0.200	0.995	862.588	1.485e +62	1.551
Cues + elasticity + cues * elasticity	0.200	0.005	0.018	6.858e +59	0.740

*Note*: All models include subject.

Because we systematically limited the information available to the observers in the No Context and Trajectory Only conditions, we hypothesized that the corresponding ratings would be noisier and that observers would be less confident in rating these stimuli. Thus, we tested whether there was a difference in the variability of responses from the same observer to repetitions of the same stimulus (intraobserver variability). We found no clear evidence for or against that in the Bayesian repeated-measures ANOVA (BF_Cue_ = 0.62, *anecdotal evidence for H_0_*). This suggests that we cannot make strong conclusions about the intraobserver variability between conditions. Presumably, more data are required in order to do so.

### Typicality ratings

**Figure 4.**
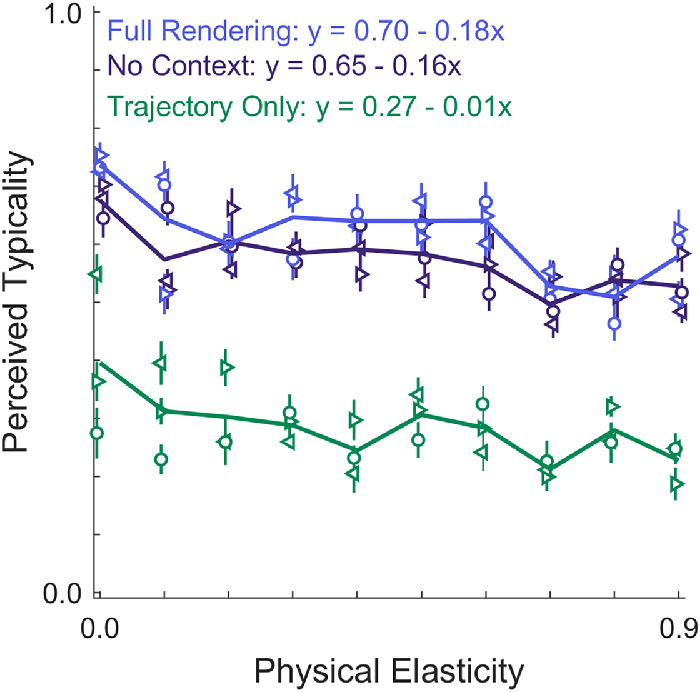
Results of the typicality ratings. Lines show average across trajectories and observers for each level of elasticity. Markers show average across observers for different trajectories (i.e., three per level of elasticity). At each elasticity level, there are three symbols of each color: The same symbol represents the same trajectory, and the color represents the cue condition (same as in Figure 3). Error bars show ± 1 *SEM* across participants. Markers of different conditions were slightly jittered along the *x*-axis to increase visibility.

Results of the typicality analysis are summarized in Table 1. We found *extreme evidence* for two independent main effects of *cue* and *elasticity* on the typicality ratings (i.e., BF_Cue + Elasticity_ = 1.45 * 10^62^). Less evidence was found for the individual main effects (BF_Elasticity_ = 1.83; BF_Cue_ = 2.98 * 10^57^). We found no evidence for an interaction effect of the two factors; that is, the data were 213.75 times more likely under the model with two main effects than a model including main and interaction effects (BF_Cue + Elasticity_ / BF_Cue + Elasticity + Interaction_ = 213.75). Thus, cue condition and elasticity had independent effects on the typicality ratings. Unsurprisingly, participants rated stimuli in which the cube rigidly followed the trajectory much less typical than stimuli in the other two cue conditions (see Figure 4). There was a smaller difference between the Full Rendering and No Context conditions (with and without background; BF_Full ≠ No Context_ = 900.2). Interestingly, we also found a mild effect of elasticity on the perceived typicality of the motion: Objects that are more elastic were perceived to be less typical. This observation is confirmed by the negative slopes that we found for a linear fit between physical elasticity and perceived typicality in all cue conditions. These results might suggest that highly elastic objects are less common in our environment; therefore, trajectories of objects low in elasticity were rated more typical. Highly elastic objects, on the other hand, might have appeared as comic-like exaggerations.

**Figure 5.**
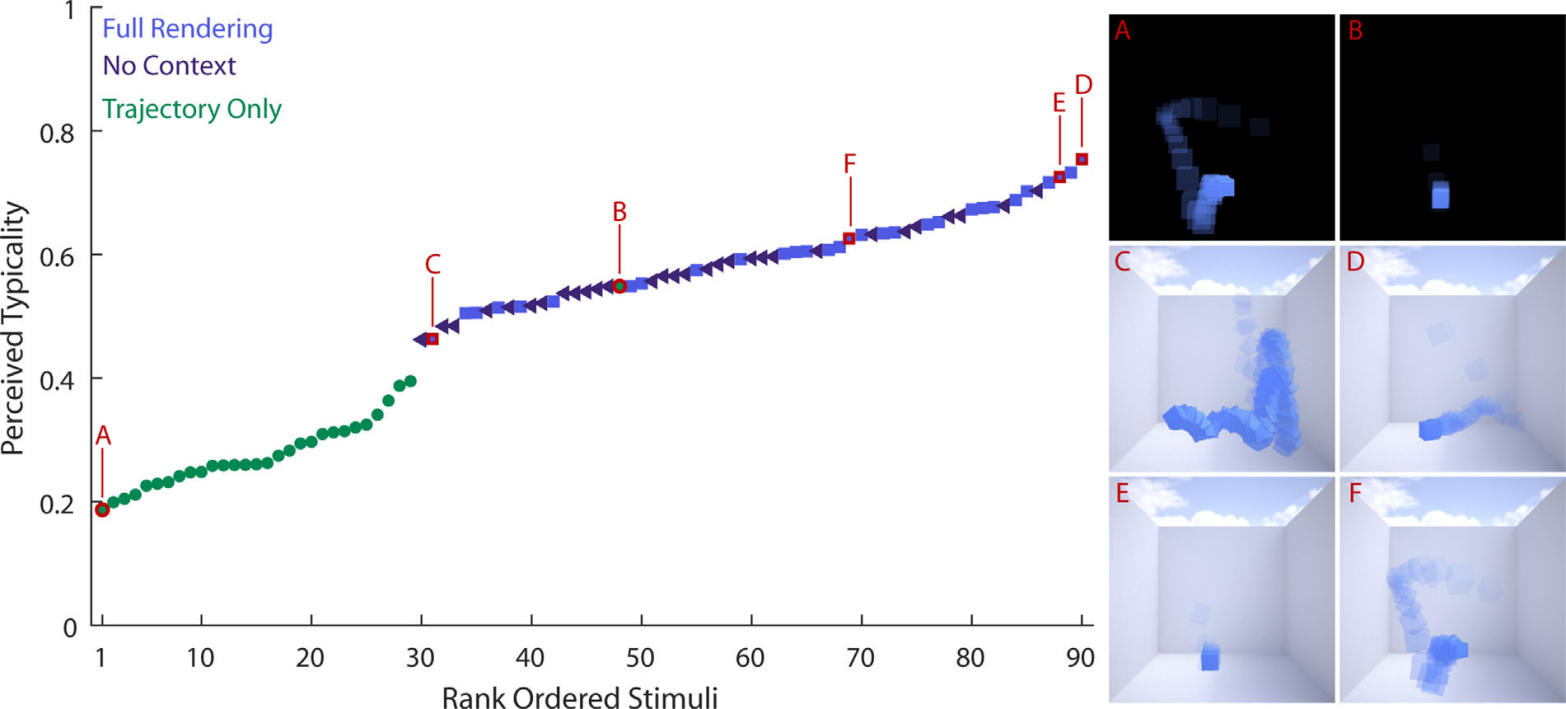
Average results of the typicality ratings rank ordered. Green circles show stimuli from the Trajectory Only condition, purple triangles show stimuli of the No Context condition, and blue squares show Full Rendering condition. Stimuli that are highlighted in red and assigned a letter are shown in Supplementary Movies S2–S4 and visualized on the right: (A) least and (B) most typical stimulus of Trajectory Only condition, (C) least and (D) most typical stimuli of Full Rendering condition, (E) most atypical stimulus of low elasticity (Full Rendering Condition), and (F) most atypical stimulus of high elasticity (Full Rendering condition).

Beside the overall effects of cue condition and elasticity on typicality, these ratings provide some additional insights when we consider individual stimuli. Figure 5 shows the stimuli rank ordered by their average typicality. Again, *Trajectory Only* stimuli were consistently rated much lower in typicality, whereas stimuli of the other two cue conditions were intermixed. It is now possible to identify the most typical (D) and the most atypical stimulus (A) over all conditions. Unsurprisingly, the least typical stimulus is highly elastic (elasticity 0.9) and of the Trajectory Only condition. Inspection of the actual stimulus shown in Supplementary Movie S2A reveals that this stimulus has a lot of movement in depth and along the line of sight of the observer. Without the walls visible, this increases the “floating” appearance of the cube in this specific exemplar, which might have added to the stimulus looking very atypical. The most typical stimulus, on the other hand, was a low elastic cube (elasticity 0.0) in the Full Rendering condition (see Supplementary Movie S3D). Figure 5 also shows that all stimuli of the Trajectory Only condition cluster at the lower end of typicality, except for one stimulus that was rated much more typical (B). Inspection of Supplementary Movie S2B demonstrates why this might be the case: The missing rotation does not stand out in this particular stimulus, because there was almost no rotation in the original trajectory. Furthermore, the cube does not bounce off any of the walls and quickly lands exactly on one of its sides, which gives a realistic impression of a flat floor. Besides the most typical stimulus of the Full Rendering condition, Supplementary Movie S3 also shows the most atypical stimulus of that condition (C): It is very elastic (elasticity 0.8) and bounces a lot. To understand better the effect that elasticity has on typicality, it is worth looking at outliers (i.e., typical elastic and atypical inelastic stimuli): Supplementary Movie S4 shows the highly elastic stimulus (elasticity 0.9) that was rated most typical (F) and the low elastic (elasticity 0.1) stimulus that was rated most atypical (E). There are at least two interesting facts to notice here. (a) The atypical low elasticity stimulus (E) indeed shows a statistically rare occurrence: The cube starts from a low position, moves upward and downward along exactly one arc, then lands exactly on one of its edges and topples over exactly once. From all possible positions the cube could land on, only six are exactly on one side; therefore, this case is statistically rare or not representative. In our set of 30 simulations, this case occurred only one time. (b) The highly elastic cube that was rated most typical in the Full Rendering condition was rated least typical (of all stimuli) in the Trajectory Only condition. Presumably, the stimulus looks less odd in the Full Rendering condition because the cube shows a lot of motion in depth and there are more depth cues available here. Why, however, does it look particularly typical? We can only speculate about this. This cube shows relatively few bounces compared to other cubes of the same elasticity, so it might have appeared less elastic. Indeed, the elasticity ratings were lower for this than for the other stimuli of the same elasticity. Therefore, what we see here might simply be the main effect of elasticity.

## Discussion

In sum, we found that observers could judge the elasticity of the bouncing cubes despite confounding variations of external factors. Participants were, however, not perfectly constant. Importantly, we found the same pattern of elasticity ratings in all three cue conditions. Stimuli in the Trajectory Only condition were generally rated slightly less elastic and their motion appeared less typical. Overall, highly elastic stimuli were rated less typical. Rated typicality also seemed to be influenced by the likelihood of observed events (e.g., how the cube lands).

Consider first the results of the Full Rendering condition. In agreement with the literature (Nusseck et al., 2007; Warren et al., 1987), we showed that observers are able to judge the elasticity of bouncing objects from visual information. Our findings extend previous reports by showing that humans can do this even if the observed motion pattern is confounded with the influence of external factors, such as a random initial velocity. However, our results also suggest that human observers do not show perfect “elasticity constancy” (i.e., they cannot perfectly discount the contribution of external factors). If they were perfectly constant, observers should have given the same ratings to different stimuli of the same elasticity level. Interestingly, these misperceptions were not random. Instead, certain stimuli were *consistently* misjudged in the same way by many participants (e.g., the same elastic stimulus was falsely rated as rather inelastic by the majority of observers). These systematic errors indicate similar underlying representations or at least the use of similar image information. By randomly varying external factors, we have created conditions that misguide the perceptual system and therefore provide potential to gain insights about the underlying mechanisms. Strikingly, stimuli that were consistently misjudged by our participants were not rated less typical. Presumably, these stimuli showed a plausible motion pattern, but one that is more typical for a different level of elasticity. Typicality judgments in the Full Rendering condition seemed to be driven by something else: (a) Stimuli appeared less typical the more elastic they were. This result is interesting, as it suggests a perceptual prior for relatively inelastic objects in the real world. (b) Stimuli also appeared less typical if they showed less likely events, such as a cube landing exactly on one side. This indicates that observers have some intuition about the statistics of the behavior of bouncing objects. Presumably, an intuition about how more or less bouncy objects *typically* behave also plays a role in the estimation of elasticity. What exactly is the information that human observers use in order to estimate the “bounciness” of an object?

In the current study, we have systematically limited the amount of visual information to gain further insights into this question. In the No Context condition, participants judged the same trajectories as in the Full Rendering condition, but they did not see the room, the floor or the walls with which the cube was interacting, or the shadow of the cube. Thus, they had less information about the bounce events and about the spatial layout in general (e.g., the depth and height of the cube). Interestingly, despite limited information, the perception of elasticity was the same as in Full Rendering condition. Observers showed the same pattern of responses here. The cube's rotation (which was visible in the No Context condition) presumably gave the observers some impression of the room. When the cube hit a wall, this became apparent through its sudden change in direction and potentially its deformation (if it was detectable). This behavior, however, not only signaled a bounce event but also indicated the presence and properties of the wall, somewhat like an illusory contour. Unsurprisingly, they rated the motion in the No Context condition as less typical than in the Full Rendering condition. Even if participants were able to make some assumptions about the room, the similarity to the ratings of the Full Rendering condition is still remarkable. This shows that the information that the visual system uses to visually infer elasticity has to be present in this condition without the background (i.e., in the mere motion trajectory).

Results of the Trajectory Only condition suggest that the deformation and rotation might add to the overall impression of elasticity but that they are not necessary for the elasticity estimate itself. This is a new and interesting finding. Previous studies (Nusseck et al., 2007; Warren et al., 1987) used undeformed spheres, whose rotations were not visible. Here, we found that the presence of both caused the motion to look more typical and overall more elastic, but the pattern of elasticity ratings, including systematic misperceptions, was the same as in the Full Rendering condition. Unlike the No Context condition, there was only a very weak impression of “illusory” walls here, because the cube's orientation stayed the same after a bounce and it did not deform when hitting a wall. This sometimes gave the cube a “floating” appearance and might have lowered the impression of elasticity. Clearly, the motion patterns in these stimuli were considered very atypical.

**Figure 6.**
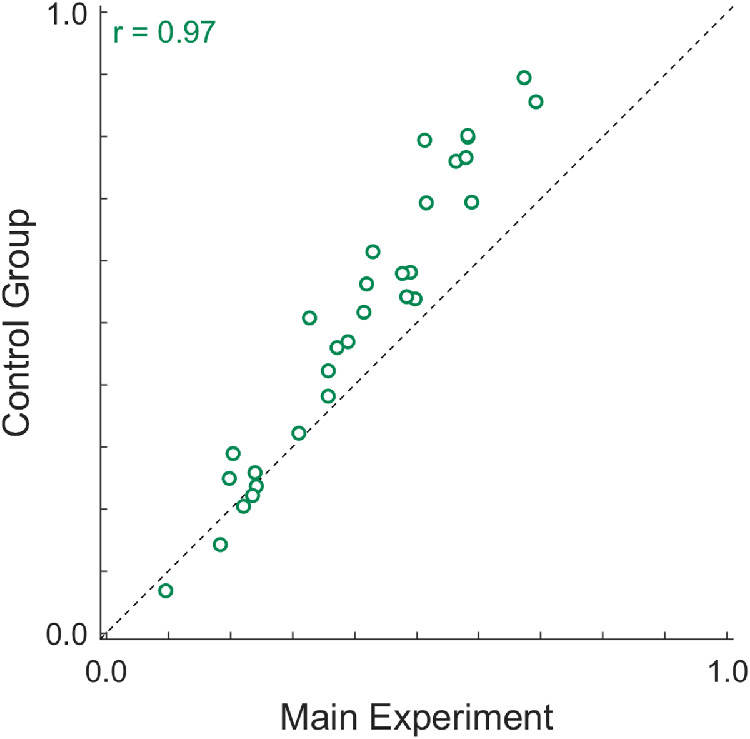
Average ratings of the control group plotted against the average ratings in the Trajectory Only condition of the main experiment. Symbols show average ratings of 30 individual stimuli. Participants of the control group had never seen the stimuli of the other two conditions of the main experiment (i.e., they had no knowledge about the layout of the scene).

One may argue that the knowledge of the room's layout from the Full Rendering condition may have helped observers to interpret the scene and estimate elasticity. The same observers saw stimuli of all conditions and thus might have assumed a similar scene layout across all conditions. In order to exclude any influence of the Full Rendering condition on the ratings in the Trajectory Only condition, we ran a small control experiment. Thirteen new participants (eight females; *M* = 23 years; *SD* = 2.24 years), none of whom had participated in the main experiment, rated the elasticity of the stimuli in the Trajectory Only condition (all other experimental details were the same as in the main experiment). As can be observed in Figure 6, we found an almost perfect correlation between the average ratings of the control group and the main experiment (*r* = .97, BF_10_ = 3.676 * 10^15^, *extreme* evidence). Highly elastic stimuli were judged slightly more elastic in the control than the main group. Presumably, this was due to the fact that observers in the control group used the same response scale for a subset of the stimuli (i.e., different anchoring of the response scale in both groups). Importantly, even without knowledge about the scene layout, observers were able to judge elasticity based only on the atypical-looking trajectories. This raises two important question: (a) If complete bounce information, rotation, and deformation are not necessary for an object to look elastic, what are the necessary conditions for an object to be judged as an elastic object at all? (b) What features of the motion trajectory make an object appear more or less elastic?

Previous research has identified several cues for elasticity estimation and suggested, for instance, that a bouncing ball looks more elastic when the square root of the ratio of the bounce height to the preceding bounce height is large (Nusseck et al., 2007; Warren et al., 1987). Warren and colleagues (1987) showed that observers could use different cues (e.g., related to the velocity) if the bounce height information was occluded. Here, we show that humans can also use a different estimation strategy if the relation between bounce height and elasticity is broken. Furthermore, Nusseck et al. (2007) showed that humans use different (or more) cues when the task changes from passive estimation to active interception. These lab results are in agreement with the great flexibility the visual system shows in the real world. The visual system seems able to adjust its strategy of estimating elasticity flexibly depending on the type of information present or the type of judgment required for the task. Based on the results of our study, we can only speculate about the trajectory features that the brain possibly uses to estimate elasticity (beside height and velocity ratios). Likely candidates for our scenes are the number of bounces, the trajectory traveled by the cube (overall or between bounces), the overall duration of the motion, or the pattern of acceleration and deceleration. Although our stimuli were simulated with the correct gravitational force, the observed gravitational constant (and therefore acceleration) was incorrect because of the size at which stimuli were presented. Interestingly, participants were able to judge relative elasticity despite the incorrect scale. Future research is necessary to test the influence of possible visual cues systematically. Presumably, these features are highly correlated and will therefore be difficult to isolate. Furthermore, future studies should not only aim at identifying visual cues but also at understanding how these cues (e.g., bounces) are computed by the visual system in the first place. For example, Jepson, Richards, and Knill (1996) derive theoretically how to detect bounces from visual information in a Bayesian inference framework, but it remains open whether human observers use the proposed strategy.

The idea that the brain uses visual features of the motion pattern in order to estimate elasticity is in line with previous research about potential mechanisms for estimating mechanical properties such as stiffness or viscosity and optical material properties such as glossiness or translucency (Kawabe et al., 2015; Kawabe & Nishida, 2016; Norman et al., 2007; Paulun et al., 2015; Paulun et al., 2017; Schmid & Doerschner, 2018; Schmidt et al., 2017; van Assen et al., 2018). Perceptual dimensions, however, might not necessarily map directly onto the physical dimensions. An important question in this regard is whether the brain tries to estimate Newtonian physics in the first place. Instead of arguing that the visual system is measuring a certain visual feature (e.g., bounce height) in order to estimate a certain physical property (e.g., elasticity), one might also argue that the brain is primarily interested in the visual feature itself (or—depending on the definition of a visual cue—a composite of features). For example, a ball will bounce high when it is highly elastic. Yet, a ball of lower elasticity might bounce to the same height when it is thrown with higher speed. One needs to disentangle these causes of a high bounce in order to estimate elasticity. However, a precise estimate of elasticity is maybe unnecessary in order to intercept the ball. Instead, the brain needs to estimate the bounce height itself, irrespective of whether elasticity or velocity contributed to it. Therefore, how high a ball bounces might be a useful estimate for a given task, although it may lead to inaccurate results when used as a proxy for elasticity. This perspective suggests that in order to understand the underlying perceptual process, it is worth looking not only for *possible* visual cues but also for *plausible* ones in terms of the tasks the brain has to solve.

## Supplementary Material

Supplement 1

Supplement 2

Supplement 3

Supplement 4

## Figures and Tables

**Figure A1.**
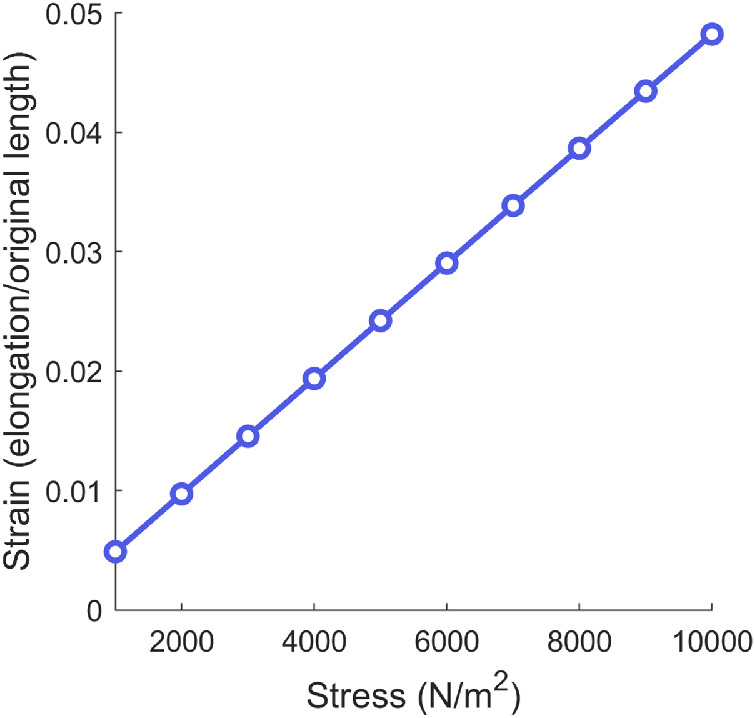
Stress-strain diagram for a cube from our experiment (elasticity = 0.5). As in the real world, there is a linear relationship between the amount of force applied and the lengthening of the material.

**Figure A2.**
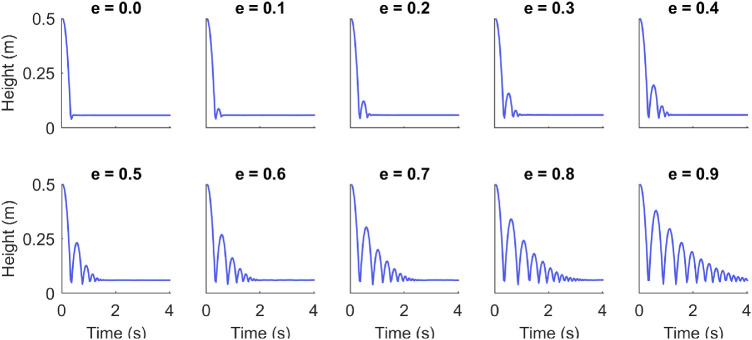
Height of spheres with different elasticities dropped from a height of 0.5 m over 4 s. Data show the height of the CoM of the spheres, which had a diameter of 0.124 m. Therefore, the height never reaches zero. The height of the first bounce was used to calculate an approximation of the coefficient of restitution. Note also that the exponential envelope of the curves (decay) is also consistent with ideal physical behavior.

**Figure A3.**
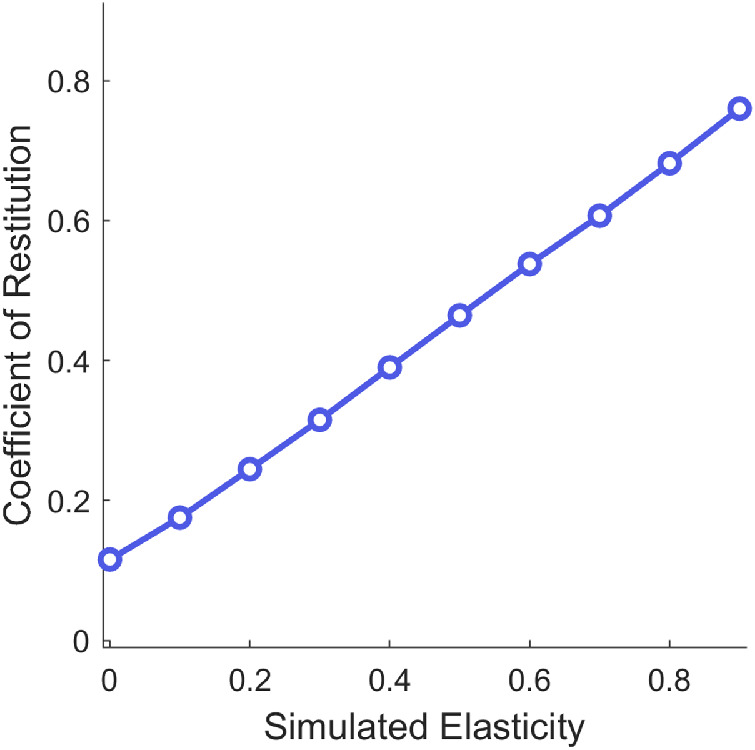
The coefficient of restitution (bounce height/drop height) for spheres of different elasticity (as defined by RealFlow's elasticity parameter).

## Appendix

### Virtual physics experiments

Any physics simulation can only approximate the complex physical behavior of objects in the real world. For our purposes, this approximation is sufficient if the macroscopic behavior of simulated objects approximately matches the macroscopic behavior in a real physical system. Qualitatively, elastic bodies must return to their original shape after an external distorting force is removed. This is true for our simulated objects, as can be seen in Figure 2, Supplementary Movie S1, and one of our previous studies using the same physics engine (Paulun et al., 2017). To quantitatively test whether the simulated macroscopic behavior is a good approximation of the real world, we further conducted two virtual experiments that make quantitative predictions about (a) the elastic modulus and (b) elastic collisions. Both were conducted with the same settings as the simulations used for the experiment (i.e., same frame rate and resolution, etc.).

#### Elastic modulus

In the real world, for small deformations, most materials show a linear relationship between stress and strain (Hooke's law). Put simply, the amount of elastic deformation increases linearly with the force applied to the material. The strength of that relationship varies between materials (i.e., the slope of the stress-strain curve). Here, we did not manipulate the elastic modulus (as we kept the stiffness parameter constant). If, however, the simulated objects realistically approximate the behavior of real elastic materials, we should find a linear relationship between stress and strain (without making a prediction about the slope of the curve). In the real world, Young's modulus is defined as the ratio of uniaxial stress or force per unit surface and the change in proportional deformation (change in length divided by original length). We measured Young's modulus of a cube of medium elasticity (e = 0.5) in RealFlow. For this purpose, the cube was attached below another cube of the same size, which was placed in the center of the room. This second cube was simulated as a “passive rigid body” (i.e., it would not deform or move under gravity but simply hold the soft cube in position). We systematically manipulated the gravity parameter to apply a unidirectional force to the soft body and measured how much it stretched under this influence. More precisely, we ran 10 simulations of 31 frames. In each simulation, the gravity value was set to a value between 10 and 100 m/s^2^ (in equal steps). We measured the elongation of the cube at each time point and compared its length in the first frame (0.1 m) to the last frame (when it had settled after some initial motion) and divided this by the original length. Figure A1 shows the resulting stress-strain curve (i.e., the elongation of the cube following linear increases in gravity). This relationship is almost perfectly linear and therefore matches the behavior of many materials in the real world.

#### Elastic collisions

In physics, collisions can be described by the proportion of kinetic energy that is retained by the system. This is quantified by the *coefficient of restitution (e)*. Accordingly, collisions can be perfectly elastic (e = 1), perfectly inelastic (e = 0), or somewhere in between. The “amount of energy that the cube retains when it collides” is exactly what is modified when changing the RealFlow parameter “elasticity,” as we did in the current study. To test whether this is indeed what the software simulates, we conducted a virtual experiment in which we dropped balls of different elasticities from the same height and measured how high they bounced. We used the ratio between the height of the first bounce and the initial height as a measure of the coefficient of restitution. We expected to find a linear increase in the measured coefficient with increasing the elasticity parameter.

To conduct the virtual physics experiment, we used the same basic scene as for the stimuli. Instead of a cube, we used a sphere, because it will not change the direction (drastically) after a bounce. The sphere was matched to the cube from the psychophysical experiment in terms of volume, density, and mesh density (important for simulation accuracy). We placed the sphere in the center of the room at 0.5 m height and let it fall freely under gravity (9.81 m/s^2^) while continuously measuring the height of the cube's CoM (at 30 fps). This was done for 10 spheres, one for each level of elasticity used in the psychophysics experiment (see Figure A2). Figure A3 shows the coefficient of restitution (ratio of first bounce height to initial height) for all levels of elasticity. Note that the coefficient is never exactly zero, because we measured the height of the CoM. Importantly, the relation between the elasticity parameter and coefficient of restitution is linear. This shows that the simulations used for the human experiments are good approximations of the real-world physics. We make no specific claims about the perception of absolute physical quantities, so the slope of the line is irrelevant for our purposes.

### Frequentist statistical analysis

In addition to the Bayesian statistics presented in this study, we will present results of the corresponding frequentist analysis in the following. More specifically, we conducted three statistical tests for potential main and interaction effects of cue condition and elasticity on the typicality as well as elasticity ratings.

1) To test for an effect of elasticity on the ratings, we used a linear fit of elasticity and typicality ratings to ground truth elasticity (irrespective of viewing condition) to determine the slope for each individual observer. We then tested whether the slopes were significantly different from zero using two-sided one-sample *t* tests. In agreement with the Bayesian analysis presented in the Results section of this article, we found that elasticity had a significant effect on the typicality as well as elasticity ratings. The slopes were significantly larger than zero in case of elasticity ratings, *t*(12) = 8.55, *p* < 0.001, and significantly smaller than zero in case of typicality ratings, *t*(12) = -2.41, *p* = 0.033.
2) To test for an effect of cue condition on both types of ratings, we compared the intercepts resulting from a linear fit of the rating data to ground truth elasticity for each cue condition individually with a one-factorial repeated-measure ANOVA. Results are summarized in Table A1. In accordance with the Bayesian analysis, we found a significant effect of cue condition on the typicality ratings. We found, however, no significant effect of cue condition on the elasticity ratings. The discrepancy to the Bayesian analysis might be due to the fact that in this ANOVA, we are comparing intercepts of linear fits (instead of raw data). Importantly, this does not affect the main conclusions of our study. The Bayesian analysis suggests the same pattern of elasticity ratings in all three cue conditions, with overall slightly lower ratings for the Trajectory Only condition. Here, we find that this difference may be negligible.
3) To test for an interaction effect of cue condition and elasticity, we compared the slopes resulting from the same linear fits of the rating data to elasticity for each cue condition with a one-factorial repeated-measure ANOVA. In accordance with the Bayesian analysis, we found no interaction effects (i.e., neither a significant difference between the slopes of the elasticity ratings nor the typicality ratings; see Table A1).

The intraobserver variability in the three viewing conditions was compared using a one-factorial repeated-measures ANOVA. In agreement with the Bayesian analysis, we found no significant effect of the cue condition on the intraobserver variability, *F*(2, 24) = 1.91, *p* = 0.170.

**Table A1.** Results of one-way repeated-measures ANOVAs on the slopes and intercepts resulting from linear fits of the rating data to ground truth elasticity for each observer.

Factor	*df*_1_	*df*_2_	*F*	*p*
*Elasticity ratings*				
Intercept (main effect of cue)	1.17	13.99	2.46	.136
Slopes (interaction Cue × Elasticity)	1.26	15.15	3.59	.070
*Typicality ratings*				
Intercept (main effect of cue)	1.30	15.62	33.20	< .001
Slopes (interaction Cue × Elasticity)	2	24	0.31	.734

*Note*: Effects were corrected for violations of sphericity using the Greenhouse-Geisser method where necessary.

Finally, the correlation between the results of the main and control experiment was 0.97 and significant at *p* < 0.001.

### Supplementary material

Supplementary Movie S1. Example trajectory in all three viewing conditions (Full Rendering, No Context and Trajectory Only). The same stimuli are visualized in the static images of Figure 1.

Supplementary Movie S2. Least (A) and most (B) typical stimulus of the Trajectory Only condition.

Supplementary Movie S3. Least (C) and most (D) typical stimulus of the Full Rendering condition.

Supplementary Movie S4. Generally, stimuli of lower elasticity appeared more typical. However, (F) shows the most typical stimulus of high elasticity (elasticity 0.9) and (E) shows the low elastic stimulus (elasticity 0.1) that was rated least typical.

## References

[bib1] Bi W., & Xiao B. (2016). Perceptual constancy of mechanical properties of cloth under variation of external forces. In Jain E. Joerg S. (Eds.), *Proceedings of the ACM Symposium on Applied Perception—SAP '16* (pp. 19–23). New York, NY: ACM.

[bib2] Brainard D. H. (1997). The psychophysics toolbox. *Spatial Vision,* 10(4), 433–436. doi:10.1163/156856897X00357 9176952

[bib3] Diaz G., Cooper J., Rothkopf C., & Hayhoe M. (2013). Saccades to future ball location reveal memory-based prediction in a virtual-reality interception task. *Journal of Vision,* 13(1), 20. doi:10.1167/13.1.20 PMC3587002 23325347

[bib4] Fleming R. W. (2014). Visual perception of materials and their properties. *Vision Research,* 94, 62–75. doi:10.1016/j.visres.2013.11.004 24291494

[bib5] Fleming R. W., Wiebel C., & Gegenfurtner K. (2013). Perceptual qualities and material classes. *Journal of Vision,* 13(8), 9. doi:10.1167/13.8.9 23847302

[bib6] Jepson A. D., Richards W., & Knill D. (1996). Modal structure and reliable inference. In *Perception as Bayesian inference* (pp. 63–92).

[bib7] Kawabe T., Maruya K., Fleming R. W., Nishida S.'Y (2015). Seeing liquids from visual motion. *Vision Research,* 109, 125–138. doi:10.1016/j.visres.2014.07.003 25102388

[bib8] Kawabe T., Nishida S.'y (2016). Seeing jelly. In Jain E. Joerg S. (Eds.), *Proceedings of the ACM Symposium on Applied Perception**—**SAP '16* (pp. 121–128). New York, NY: ACM.

[bib9] Kersten D., Mamassian P., & Knill D. C. (1997). Moving cast shadows induce apparent motion in depth. *Perception,* 26, 171–192. doi:10.1068/p260171 9274752

[bib10] Kleiner M., Brainard D., Pelli D., Ingling A., Nurray R., & Broussard C. (2007). What's new in Psychtoolbox-3. *Perception,* 36, 1.

[bib11] Masuda T., Sato K., Murakoshi T., Utsumi K., Kimura A., Shirai N., … (2013). Perception of elasticity in the kinetic illusory object with phase differences in inducer motion. *PLoS One,* 8, e78621. doi:10.1371/journal.pone.0078621 24205281 PMC3808284

[bib12] Norman J. F., Wiesemann E. Y., Norman H. F., Taylor M. J., & Craft W. D. (2007). The visual discrimination of bending. *Perception,* 36, 980–989. doi:10.1068/p5641 17844964

[bib13] Nusseck M., Lagarde J., Bardy B., Fleming R., & Bülthoff H. H. (2007). Perception and prediction of simple object interactions. In Wallraven C. Sundstedt V. (Eds.), *Proceedings of the 4th Symposium on Applied Perception in Graphics and Visualization* (p. 27). New York, NY: ACM.

[bib14] Paulun V. C., Gegenfurtner K. R., Goodale M. A., & Fleming R. W. (2016). Effects of material properties and object orientation on precision grip kinematics. *Experimental Brain Research,* 234, 2253–2265. doi:10.1007/s00221-016-4631-7 27016090 PMC4923101

[bib15] Paulun V. C., Kawabe T., Nishida S.'y., & Fleming R. W. (2015). Seeing liquids from static snapshots. *Vision Research,* 115(Pt B), 163–174. doi:10.1016/j.visres.2015.01.023 25676882

[bib16] Paulun V. C., Schmidt F., van Assen J. J. R., & Fleming R. W. (2017). Shape, motion, and optical cues to stiffness of elastic objects. *Journal of Vision,* 17(1), 20. doi:10.1167/17.1.20 28114494

[bib17] Pelli D. G. (1997). The VideoToolbox software for visual psychophysics: transforming numbers into movies. *Spatial Vision,* 10, 437–442. doi:10.1163/156856897X00366 9176953

[bib18] Schmid A. C., & Doerschner K. (2018). Shatter and splatter: The contribution of mechanical and optical properties to the perception of soft and hard breaking materials. *Journal of Vision,* 18(1), 14. doi:10.1167/18.1.14 29362807

[bib19] Schmidt F., Paulun V. C., van Assen J. J. R., & Fleming R. W. (2017). Inferring the stiffness of unfamiliar objects from optical, shape, and motion cues. *Journal of Vision,* 17(3), 18. doi:10.1167/17.3.18 28355630

[bib20] Twardy C. R., & Bingham G. P. (2002). Causation, causal perception, and conservation laws. *Perception & Psychophysics,* 64, 956–968. 12269302 10.3758/bf03196799

[bib21] van Assen J. J. R., Barla P., & Fleming R. W. (2018). Visual features in the perception of liquids. *Current Biology,* 28, 452–458.e4. doi:10.1016/j.cub.2017.12.037 29395924 PMC5807092

[bib22] Wagenmakers E.-J., Love J., Marsman M., Jamil T., Ly A., Verhagen J., … (2018). Bayesian inference for psychology. Part II: Example applications with JASP. *Psychonomic Bulletin & Review,* 25, 58–76. doi:10.3758/s13423-017-1323-7 28685272 PMC5862926

[bib23] Warren W. H., Kim E. E., & Husney R. (1987). The way the ball bounces: visual and auditory perception of elasticity and control of the bounce pass. *Perception,* 16, 309–336. doi:10.1068/p160309 3432028

